# Supplementary material for: The Magnitude of Cardiovascular Disease Risk Factors in Seafarers from 1994 to 2021: A Systematic Review and Meta-Analysis
Source: J Pers Med. 2023 May 20;13(5):861. doi: 10.3390/jpm13050861 (PMC10224038; doi:10.3390/jpm13050861)
Supplement: Supplementary file 1 [file jpm-13-00861-s001.zip › Supplementary Figure.pdf]

Supplementary Figure:

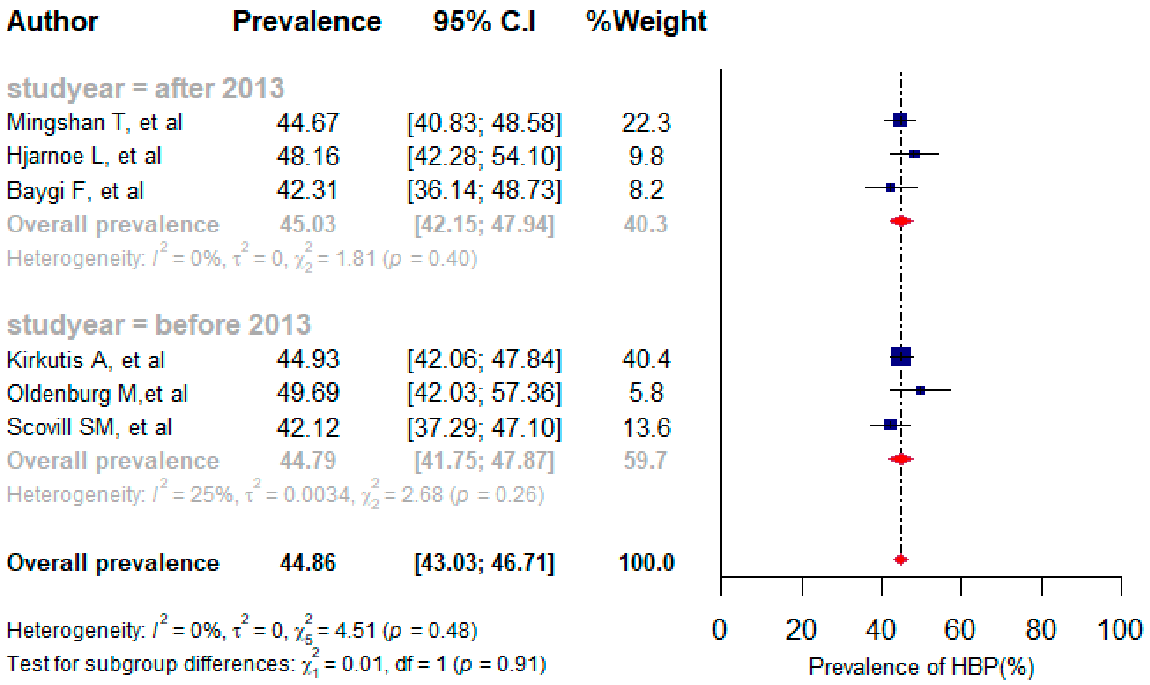

Supplementary Figure 1. A forest plot of the prevalence (%) of high blood pressure among seafarers after omitting the outlier studies.

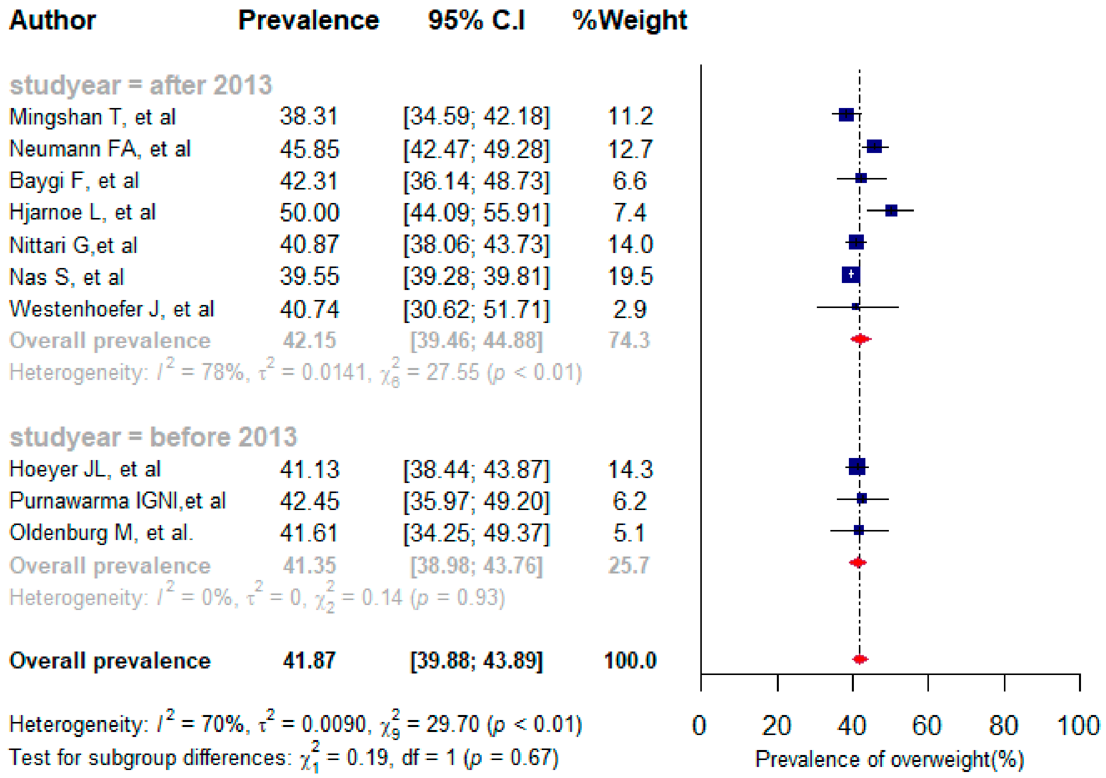

Supplementary Figure 2. A forest plot of the prevalence (%) of overweight among seafarers after omitting the outlier studies.

Author	Prevalence	95% C.I	%Weight
**studyear = after 2013**			
Mingshan T, et al	17.33	[14.57; 20.49]	9.7
Neumann FA, et al	9.76	[7.91; 11.98]	9.6
Baygi F, et al	8.55	[5.58; 12.88]	7.7
Hjarnoe L, et al	25.00	[20.21; 30.49]	9.3
Nittari G, et al	11.26	[9.56; 13.21]	9.9
Nas S, et al	12.55	[12.37; 12.73]	10.4
Westenhoefer J, et al	34.57	[25.05; 45.51]	7.8
Overall prevalence	15.14	[11.93; 19.03]	64.3
Heterogeneity: $I^2 = 93\%$, $\tau^2 = 0.1181$, $\chi^2_6 = 90.97$ ($p < 0.01$)			
**studyear = before 2013**			
Hoeyer JL, et al	22.91	[20.67; 25.32]	10.1
Purnawarma IGNI, et al	10.38	[6.93; 15.26]	7.9
Hansen HL, et al	16.05	[12.56; 20.28]	9.2
Oldenburg M, et al	21.74	[16.04; 28.77]	8.5
Overall prevalence	17.57	[12.73; 23.74]	35.7
Heterogeneity: $I^2 = 86\%$, $\tau^2 = 0.1233$, $\chi^2_3 = 21.44$ ($p < 0.01$)			
**Overall prevalence**	**15.99**	**[12.88; 19.68]**	**100.0**

Heterogeneity: $I^2 = 95\%$, $\tau^2 = 0.1595$, $\chi^2_{10} = 221.54$ ($p < 0.01$)
Test for subgroup differences: $\chi^2_1 = 0.55$, df = 1 ($p = 0.46$)

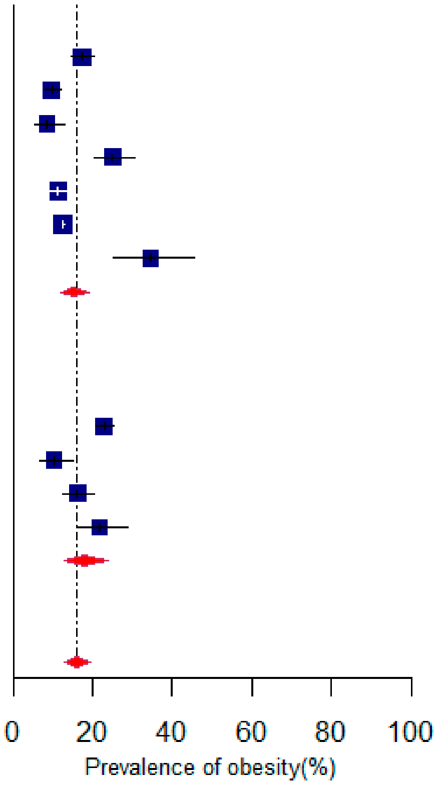

Supplementary Figure 3. A forest plot of the prevalence (%) of obesity among seafarers after omitting the outlier studies.

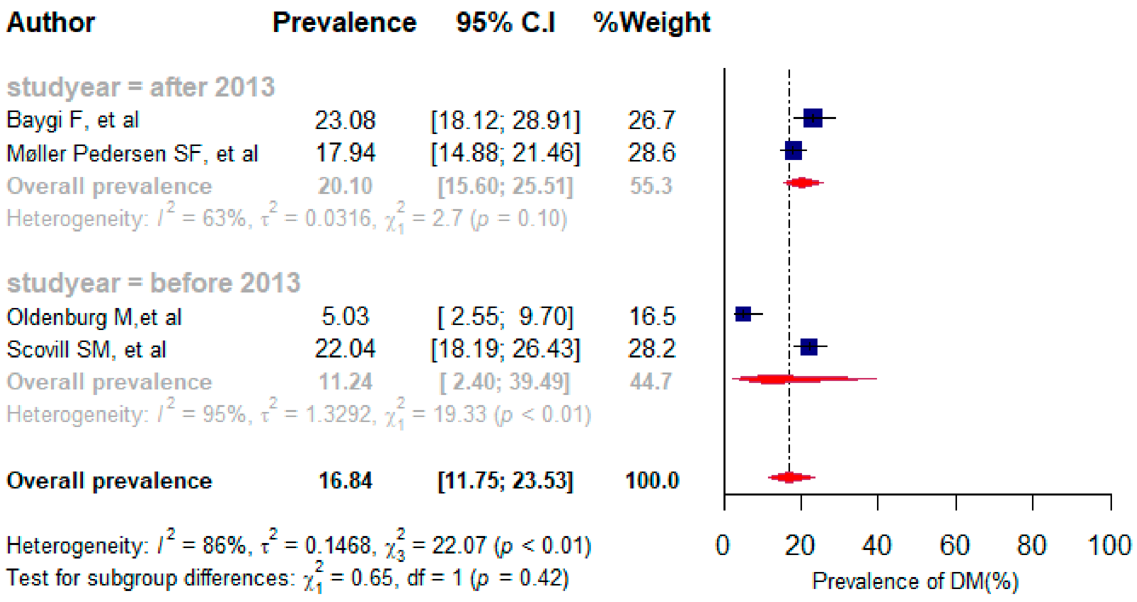

Supplementary Figure 4. A forest plot of the prevalence (%) of diabetes mellitus among seafarers after omitting the outlier studies.

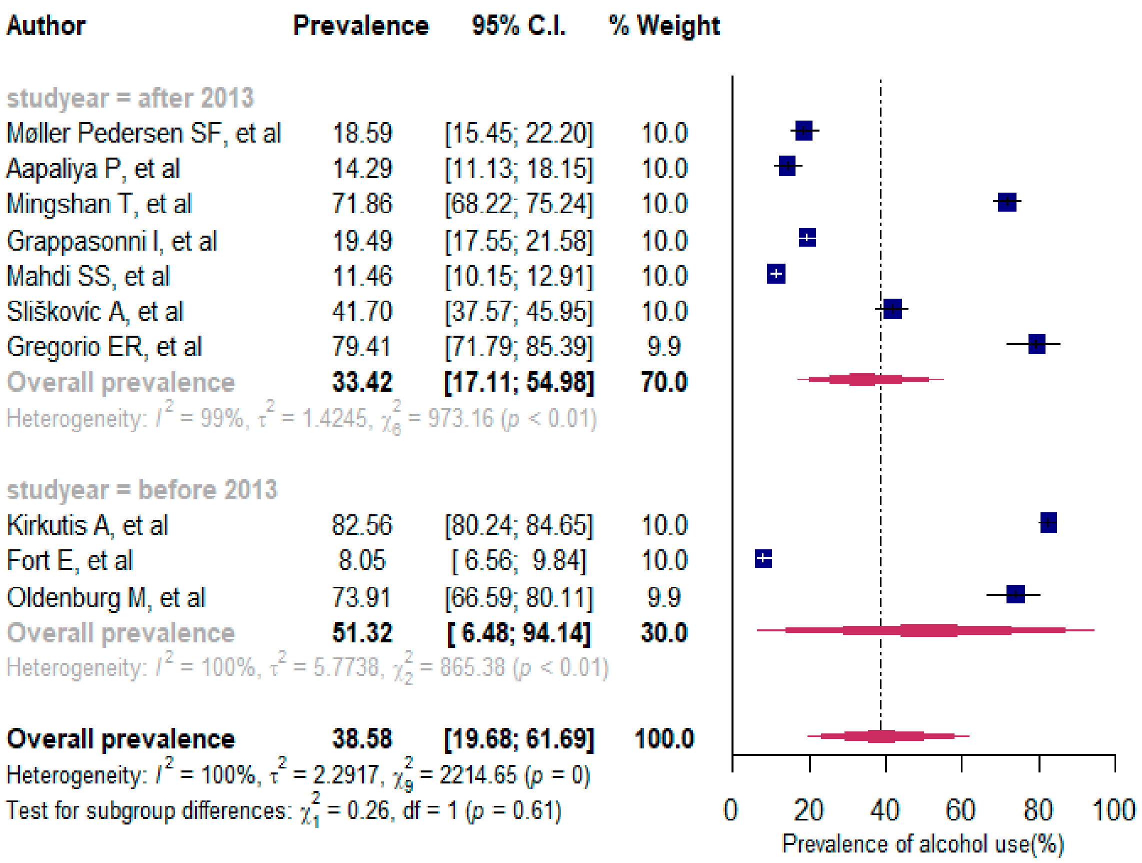

Supplementary Figure 5. A forest plot of the prevalence (%) of alcohol use among seafarers using a random effect model.
