# Supplementary material for: The Magnitude of Cardiovascular Disease Risk Factors in Seafarers from 1994 to 2021: A Systematic Review and Meta-Analysis
Source: J Pers Med. 2023 May 20;13(5):861. doi: 10.3390/jpm13050861 (PMC10224038; doi:10.3390/jpm13050861)
Supplement: Supplementary file 1 [file jpm-13-00861-s001.zip › Supplementary Table.pdf]

Supplementary Table:

Supplementary Table 1. Literature Search strategies and results

Database	Themes	Search key words and combinations	Results
PubMed	High blood pressure	(((("Prevalence"[Title/Abstract] OR "proportion"[Title/Abstract] OR "magnitude"[Title/Abstract]) AND "high blood pressure"[Title/Abstract]) OR "hypertension"[Title/Abstract]) AND "seafarers"[Title/Abstract]) OR "sailing seafarers"[Title/Abstract] OR "onboard ships"[Title/Abstract] OR "merchant ships"[Title/Abstract]	144
	Overweight	(((("Prevalence"[Title/Abstract] OR "proportion"[Title/Abstract] OR "magnitude"[Title/Abstract]) AND "overweight"[Title/Abstract]) OR "high body mass index"[Title/Abstract]) AND "seafarers"[Title/Abstract]) OR "sailing seafarers"[Title/Abstract] OR "onboard ships"[Title/Abstract] OR "merchant ships"[Title/Abstract]	133
	Obesity	(((("Prevalence"[Title/Abstract] OR "proportion"[Title/Abstract] OR "magnitude"[Title/Abstract]) AND "obesity"[Title/Abstract]) OR "high body mass index"[Title/Abstract]) AND "seafarers"[Title/Abstract]) OR "sailing seafarers"[Title/Abstract] OR "onboard ships"[Title/Abstract] OR "merchant ships"[Title/Abstract]	141
	Smoking	(((("Prevalence"[Title/Abstract] OR "proportion"[Title/Abstract] OR "magnitude"[Title/Abstract]) AND "smoking"[Title/Abstract]) OR "cigarette smoking"[Title/Abstract] OR "tobacco"[Title/Abstract] OR "tobacco smoking"[Title/Abstract]) AND "seafarers"[Title/Abstract]) OR "sailing seafarers"[Title/Abstract] OR "onboard ships"[Title/Abstract] OR "merchant ships"[Title/Abstract]	152

	Diabetes mellitus	(((("Prevalence"[Title/Abstract] OR "proportion"[Title/Abstract] OR "magnitude"[Title/Abstract]) AND "diabetes"[Title/Abstract]) OR "diabetes mellitus"[Title/Abstract] OR "blood glucose level"[Title/Abstract]) AND "seafarers"[Title/Abstract]) OR "sailing seafarers"[Title/Abstract] OR "onboard ships"[Title/Abstract] OR "merchant ships"[Title/Abstract]	136
	Alcohol consumption	(((("prevalence"[Title/Abstract] OR "proportion"[Title/Abstract] OR "magnitude"[Title/Abstract]) AND "alcohol"[Title/Abstract]) OR "alcoholism"[Title/Abstract] OR "alcoholate"[Title/Abstract] OR "alcoholic"[Title/Abstract] OR "alcoholics"[Title/Abstract]) AND "seafarers"[Title/Abstract]) OR "sailing seafarers"[Title/Abstract] OR "onboard ships"[Title/Abstract] OR "merchant ships"[Title/Abstract]	135
Scopus	High blood pressure	TITLE-ABS KEY ("prevalence" OR "proportion" OR "magnitude" AND "high bloodpressure" OR "hypertension" AND "seafarers" OR "sailors" OR "onboard ships" OR "merchant ships")	25
	Overweight	TITLE-ABS KEY ("prevalence" OR "proportion" OR "magnitude" AND "overweight" OR "high body mass index" AND "seafarers" OR "sailors" OR "onboard ships" OR "merchant ships")	13
	Obesity	TITLE-ABS KEY ("prevalence" OR "proportion" OR "magnitude" AND "obesity" OR "high body mass index" AND "seafarers" OR "sailors" OR "onboard ships" OR "merchant ships")	24
	Smoking	TITLE-ABS KEY ("prevalence" OR "proportion" OR "magnitude" AND "smoking" OR "cigarette smoking" OR "tobacco" OR "tobacco smoking" AND "seafarers" OR "sailors" OR "onboard ships" OR "merchant ships")	38
	Diabetes mellitus	TITLE-ABS-KEY ("prevalence" OR "proportion" OR "magnitude" AND "diabetes" OR "diabetes mellitus" OR "blood glucose level" AND "seafarers" OR "sailors" OR "onboard ships" OR "merchant ships")	15

	Alcohol consumption	TITLE-ABS KEY ("prevalence" OR "proportion" OR "magnitude" AND "alcohol" OR "alcoholism" OR "alcoholic" OR "alcoholate" OR "alcoholics" AND "seafarers" OR "sailing" OR "onboard OR "ships" OR "vessels")	13

Supplementary Table 2. The Joanna Briggs Institute (JBI) Prevalence Critical Appraisal Tool

S.No	Criteria	Yes	No	Unclear	Not applicable
1	Was the sample representative of the target population?				
2	Were study participants recruited in an appropriate way?				
3	Was the sample size adequate?				
4	Were the study subjects and the setting described in detail?				
5	Was the data analysis conducted with sufficient coverage of the identified sample?				
6	Were objective, standard criteria used for the measurement of the condition?				
7	Was the condition measured reliably?				
8	Was there appropriate statistical analysis?				
9	Are all important confounding factors/subgroups/differences identified and accounted for?				
10	Were subpopulations identified using objective criteria?				

Supplementary Table 3. Leave-one-out analysis for prevalence of smoking in seafarers.

	estimate	zval	pval	ci.lb	ci.ub	Q	Qp	tau2	I2	H2
1	0.4082	-2.8194	0.0048	0.3477	0.4717	573.7180	0	0.2485	97.5598	40.9799
2	0.4124	-2.7480	0.0060	0.3528	0.4746	559.1361	0	0.2378	97.4961	39.9383
3	0.4139	-2.7860	0.0053	0.3560	0.4742	513.9905	0	0.2223	97.2762	36.7136
4	0.4098	-2.9221	0.0035	0.3522	0.4700	472.8460	0	0.2218	97.0392	33.7747
5	0.3913	-3.3370	0.0008	0.3315	0.4546	538.1938	0	0.2509	97.3987	38.4424
6	0.3907	-3.5326	0.0004	0.3339	0.4507	445.0794	0	0.2252	96.8545	31.7914
7	0.3983	-2.8362	0.0046	0.3324	0.4682	608.5814	0	0.3051	97.6996	43.4701
8	0.4099	-2.7776	0.0055	0.3496	0.4732	586.8265	0	0.2469	97.6143	41.9162
9	0.4007	-2.8806	0.0004	0.3370	0.4679	606.8214	0	0.2811	97.6929	43.3444
10	0.3989	-3.0436	0.0023	0.3376	0.4636	609.4842	0	0.2609	97.7030	43.5346
11	0.4032	-2.9506	0.0032	0.3424	0.4671	607.0490	0	0.2545	97.6938	43.3606
12	0.3966	-3.1410	0.0017	0.3359	0.4606	607.9238	0	0.2567	97.6971	43.4231
13	0.4007	-2.9814	0.0029	0.3391	0.4656	608.6608	0	0.2619	97.6999	43.4758
14	0.4000	-2.9770	0.0029	0.3380	0.4654	609.1341	0	0.2664	97.7017	43.5096
15	0.3838	-3.7830	0.0002	0.3276	0.4432	523.7089	0	0.2237	97.3268	37.4078
16	0.4039	-2.9340	0.0033	0.3432	0.4678	606.5165	0	0.2536	97.6917	43.3226

Supplementary Table 4. Leave-one-out diagnostics with a built-in function in smoking prevalence among seafarers.

	rstudent	dffits	cook.d	cov.r	tau2.del	QE.del	hat	weight	dfbs	inf
1	-0.8597	-0.2233	0.0495	1.0584	0.2485	573.7180	0.0631	6.3085	-0.2233	
2	-1.4177	-0.3656	0.1272	1.0134	0.2378	559.1361	0.0619	6.1944	-0.3657	
3	-1.6373	-0.4293	0.1641	0.9517	0.2223	513.9905	0.0631	6.3144	-0.4290	
4	-1.0857	-0.2900	0.0745	0.9512	0.2218	472.8460	0.0645	6.4503	-0.2895	
5	1.2506	0.3282	0.1078	1.0697	0.2509	538.1938	0.0644	6.4433	0.3282	
6	1.4001	0.3669	0.1210	0.9653	0.2252	445.0794	0.0648	6.4839	0.3662	
7	0.3269	0.0900	0.0098	1.2906	0.3051	608.5814	0.0648	6.4782	0.0903	
8	-1.1071	-0.2805	0.0776	1.0486	0.2469	586.8265	0.0602	6.0231	-0.2806	
9	0.0709	0.0214	0.0005	1.1924	0.2811	606.8214	0.0644	6.4355	0.0214	
10	0.3041	0.0795	0.0066	1.1081	0.2609	609.4842	0.0623	6.2335	0.0795	
11	-0.2353	-0.0586	0.0035	1.0783	0.2545	607.0490	0.0591	5.9087	-0.0586	
12	0.6003	0.1534	0.0241	1.0891	0.2567	607.9238	0.0608	6.0766	0.1534	
13	0.0780	0.0213	0.0005	1.1125	0.2619	608.6608	0.0627	6.2680	0.0213	
14	0.1584	0.0428	0.0019	1.1317	0.2664	609.1341	0.0634	6.3391	0.0428	
15	2.3750	0.6093	0.3331	0.9566	0.2237	523.7089	0.0625	6.2452	0.6094	
16	-0.3362	-0.0832	0.0070	1.0731	0.2536	606.5165	0.0580	5.7972	-0.0831	

Supplementary Table 5. Leave-one-out analysis for prevalence of high blood pressure in seafarers.

	estimate	zval	pval	ci.lb	ci.ub	Q	Qp	tau2	I2	H2
1	0.4536	-0.8738	0.3822	0.3535	0.5576	164.4557	0	0.3025	96.3516	27.4093
2	0.4489	-1.0275	0.3042	0.3551	0.5464	166.2604	0	0.2652	96.3912	27.7101
3	0.4182	-3.0000	0.0027	0.3669	0.4714	45.7007	0	0.0704	86.8711	7.6168
4	0.4572	-0.8724	0.3830	0.3643	0.5532	164.0072	0	0.2566	96.3416	27.3345
5	0.4530	-0.8352	0.4036	0.3472	0.5632	162.9707	0	0.3412	96.3184	27.1618
6	0.4471	-1.0921	0.2748	0.3558	0.5421	165.9465	0	0.2522	96.3844	27.6577
7	0.4908	-0.2302	0.8179	0.4131	0.5689	113.2739	0	0.1678	94.7031	18.8790
8	0.4575	-0.8443	0.3985	0.3623	0.5560	161.9363	0	0.2699	96.2948	26.9894

Supplementary Table 6. Leave-one-out diagnostics with a built-in function in high blood pressure among seafarers.

	rstudent	dffits	cook.d	cov.r	tau2.del	QE.del	hat	weight	dfbs	inf
1	-0.0465	-0.0082	0.0001	1.4623	0.3025	164.4557	0.1290	12.8991	-0.0083	
2	0.2328	0.0931	0.0097	1.2849	0.2652	166.2604	0.1247	12.4694	0.0931	
3	3.9542	1.4117	0.6517	0.3899	0.0704	45.7007	0.1275	12.7544	1.3876	*
4	-0.2481	-0.0892	0.0087	1.2437	0.2566	164.0072	0.1233	12.3316	-0.0892	
5	-0.0234	0.0036	0	1.6424	0.3412	162.9707	0.1305	13.0548	0.0036	
6	0.3565	0.1352	0.0195	1.2190	0.2522	165.9465	0.1198	11.9824	0.1350	
7	-2.7068	-0.9913	0.7337	0.8279	0.1678	113.2739	0.1183	11.8288	-1.0012	*
8	-0.2605	-0.0933	0.0100	1.3095	0.2699	161.9363	0.1268	12.6796	-0.0933	

Supplementary Table 7. Leave-one-out analysis for prevalence of overweight in seafarers.

	estimate	zval	pval	ci.lb	ci.ub	Q	Qp	tau2	I2	H2
1	0.4203	-5.4943	0	0.3927	0.4485	70.7570	0	0.0261	85.8671	7.0757
2	0.4119	-6.4982	0	0.3862	0.4382	57.9860	0	0.0216	82.7545	5.7986
3	0.4162	-6.0279	0	0.3898	0.4432	70.5263	0	0.0242	85.8209	7.0526
4	0.4100	-6.9552	0	0.3855	0.4351	59.1779	0	0.0198	83.1018	5.9178
5	0.4178	-5.4164	0	0.3889	0.4473	70.4925	0	0.0291	85.8141	7.0493
6	0.4197	-4.5194	0	0.3859	0.4542	59.4419	0	0.0433	83.1769	5.9442
7	0.4171	-6.1067	0	0.3912	0.4434	71.1809	0	0.0238	85.9513	7.1181
8	0.4175	-5.3999	0	0.3884	0.4472	70.0482	0	0.0296	85.7241	7.0048
9	0.4161	-6.0531	0	0.3898	0.4430	70.5214	0	0.0241	85.8199	7.0521
10	0.4284	-5.8995	0	0.4051	0.4520	50.0223	0	0.0163	80.0089	5.0022
11	0.4079	-7.5392	0	0.3847	0.4315	50.7933	0	0.0167	80.3124	5.0793
12	0.4167	-6.0421	0	0.3905	0.4435	70.9597	0	0.0240	85.9075	7.0960

Supplementary Table 8. Leave-one-out diagnostics with a built-in function in overweight prevalence among seafarers.

	rstudent	dffits	cook.d	cov.r	tau2.del	QE.del	hat	weight	dfbs	inf
1	-0.8125	-0.2693	0.0800	1.2108	0.0261	70.7570	0.0949	9.4897	-0.2700	
2	1.1045	0.3774	0.1351	1.0618	0.0216	57.9860	0.1011	10.1053	0.3763	
3	0.1329	0.0335	0.0012	1.1141	0.0242	70.5263	0.0697	6.9679	0.0333	
4	1.8844	0.5369	0.2636	0.9677	0.0198	59.1779	0.0748	7.4849	0.5422	
5	-0.1972	-0.0789	0.0076	1.3277	0.0291	70.4925	0.1061	10.6094	-0.0801	
6	-0.4552	-0.1699	0.0541	1.8193	0.0433	59.4419	0.1224	12.2405	-0.1825	
7	-0.1428	-0.0308	0.0010	1.0635	0.0238	71.1809	0.0381	3.8100	-0.0306	
8	-0.1335	-0.0584	0.0043	1.3453	0.0296	70.0482	0.1073	10.7290	-0.0594	
9	0.1596	0.0401	0.0016	1.1071	0.0241	70.5214	0.0667	6.6720	0.0400	
10	-3.6755	-1.0045	0.8176	0.8454	0.0163	50.0223	0.0789	7.8918	-1.0231	*
11	2.4903	0.7525	0.4618	0.8641	0.0167	50.7933	0.0819	8.1856	0.7621	*
12	-0.0105	-0.0054	0	1.0952	0.0240	70.9597	0.0581	5.8137	-0.0054	

Supplementary Table 9. Leave-one-out analysis for prevalence of obesity in seafarers.

	estimate	zval	pval	ci.lb	ci.ub	Q	Qp	tau2	I2	H2
1	0.1871	-6.4865	0	0.1287	0.2641	727.9867	0	0.5396	98.6263	72.7987
2	0.1967	-6.2767	0	0.1362	0.2753	731.8109	0	0.5289	98.6335	73.1811
3	0.1978	-6.4731	0	0.1389	0.2736	735.0973	0	0.4942	98.6396	73.5097
4	0.1808	-6.9609	0	0.1260	0.2524	705.2348	0	0.4949	98.5820	70.5235
5	0.1944	-6.1353	0	0.1328	0.2754	736.1966	0	0.5662	98.6417	73.6197
6	0.1925	-5.5758	0	0.1259	0.2830	470.2181	0	0.7016	97.8733	47.0218
7	0.1754	-7.2801	0	0.1230	0.2440	709.2682	0	0.4767	98.5901	70.9268
8	0.1822	-6.4377	0	0.1236	0.2603	629.4393	0	0.5735	98.4113	62.9439
9	0.1950	-6.5360	0	0.1367	0.2704	737.7781	0	0.4970	98.6446	73.7778
10	0.1599	-12.8789	0	0.1288	0.1968	221.5430	0	0.1595	95.4862	22.1543
11	0.1884	-6.6075	0	0.1309	0.2636	735.8840	0	0.5139	98.6411	73.5884
12	0.1834	-6.8881	0	0.1280	0.2556	728.0904	0	0.4953	98.6265	72.8090

Supplementary Table 10. Leave-one-out diagnostics with a built-in function in obesity prevalence among seafarers.

	rstudent	dffits	cook.d	cov.r	tau2.del	QE.del	hat	weight	dfbs	inf
1	-0.1209	-0.0355	0.0014	1.2114	0.5396	727.9867	0.0854	8.5361	-0.0355	
2	-1.0612	-0.3216	0.1126	1.1879	0.5289	731.8109	0.0849	8.4889	-0.3219	
3	-1.2567	-0.3669	0.1370	1.1056	0.4942	735.0973	0.0785	7.8465	-0.3667	
4	0.5504	0.1668	0.0284	1.1134	0.4949	705.2348	0.0839	8.3920	0.1669	
5	-0.8111	-0.2456	0.0703	1.2690	0.5662	736.1966	0.0858	8.5781	-0.2461	
6	-0.5795	-0.1730	0.0433	1.5619	0.7016	470.2181	0.0873	8.7302	-0.1742	
7	1.1980	0.3490	0.1200	1.0678	0.4767	709.2682	0.0785	7.8478	0.3492	
8	0.3626	0.1129	0.0151	1.2856	0.5735	629.4393	0.0865	8.6511	0.1133	
9	-0.9568	-0.2803	0.0804	1.1122	0.4970	737.7781	0.0790	7.9044	-0.2802	
10	4.8801	1.5138	0.7876	0.3921	0.1595	221.5430	0.0854	8.5405	1.4848	*
11	-0.2545	-0.0761	0.0061	1.1541	0.5139	735.8840	0.0836	8.3645	-0.0761	
12	0.2797	0.0835	0.0071	1.1112	0.4953	728.0904	0.0812	8.1199	0.0835	

Supplementary Table 11. Leave-one-out analysis for prevalence of diabetes mellitus in seafarers.

	estimate	zval	pval	ci.lb	ci.ub	Q	Qp	tau2	I2	H2
1	0.1029	-6.1060	0	0.0541	0.1869	42.9332	0	0.4344	93.0124	14.3111
2	0.1079	-5.0890	0	0.0509	0.2144	46.9716	0	0.6149	93.6132	15.6572
3	0.1558	-6.7351	0	0.1014	0.2318	30.2618	0	0.2117	90.0865	10.0873
4	0.1025	-5.6180	0	0.0508	0.1958	42.1483	0	0.5241	92.8823	14.0494
5	0.1684	-7.4756	0	0.1175	0.2353	22.0728	0	0.1468	86.4086	7.3576

Supplementary Table 12. Leave-one-out diagnostics with a built-in function in diabetes mellitus prevalence among seafarers.

	rstudent	dffits	cook.d	cov.r	tau2.del	QE.del	hat	weight	dfbs	inf
1	1.2581	0.7506	0.7708	1.7088	0.4344	42.9332	0.2197	21.9663	0.7582	
2	0.6612	0.4885	0.4624	2.3395	0.6149	46.9716	0.2272	22.7195	0.5018	
3	-1.9621	-0.9951	0.7672	0.8551	0.2117	30.2618	0.1669	16.6910	-1.0308	
4	1.0924	0.6949	0.7983	2.0264	0.5241	42.1483	0.2258	22.5775	0.7092	
5	-3.0517	-1.5191	1.4814	0.6201	0.1468	22.0728	0.1605	16.0457	-1.6611	*

Supplementary Table 13. Leave-one-out analysis for prevalence of alcohol consumption in seafarers.

	estimate	zval	pval	ci.lb	ci.ub	Q	Qp	tau2	I2	H2
1	0.4128	-0.6735	0.5006	0.2013	0.6622	2161.3508	0	2.4488	99.6299	270.1689
2	0.4211	-0.6198	0.5354	0.2101	0.6655	2153.3648	0	2.3560	99.6285	269.1706
3	0.3494	-1.2519	0.2106	0.1686	0.5870	1841.0286	0	2.2039	99.5655	230.1286
4	0.4114	-0.6491	0.5163	0.1916	0.6733	2053.1664	0	2.7240	99.6104	256.6458
5	0.4283	-0.5753	0.5651	0.2188	0.6671	1732.8530	0	2.2518	99.5383	216.6066
6	0.3824	-0.8825	0.3775	0.1760	0.6422	2197.7499	0	2.6375	99.6360	274.7187
7	0.3398	-1.3233	0.1857	0.1614	0.5792	2121.5515	0	2.2544	99.6229	265.1939
8	0.3334	-1.6516	0.0986	0.1802	0.5323	1262.2314	0	1.5668	99.3662	157.7789
9	0.4388	-0.4945	0.6210	0.2276	0.6747	1951.2399	0	2.2142	99.5900	243.9050
10	0.3472	-1.2514	0.2108	0.1652	0.5884	2120.4848	0	2.2767	99.6227	265.0606

Supplementary Table 14. **Leave-one-out diagnostics with a built-in function in alcohol consumption prevalence in seafarers.**

	rstudent	dffits	cook.d	cov.r	tau2.del	QE.del	hat	weight	dfbs	inf
1	-0.6798	-0.2271	0.0551	1.1869	2.4488	2161.3508	0.1001	10.0108	-0.2271	
2	-0.9068	-0.3021	0.0938	1.1418	2.3560	2153.3648	0.0997	9.9749	-0.3021	
3	0.9945	0.3324	0.1063	1.0692	2.2039	1841.0286	0.1003	10.0328	0.3324	
4	-0.6090	-0.2044	0.0496	1.3199	2.7240	2053.1664	0.1005	10.0483	-0.2044	
5	-1.1088	-0.3704	0.1348	1.0925	2.2518	1732.8530	0.1005	10.0463	-0.3704	
6	0.0840	0.0272	0.0009	1.2781	2.6375	2197.7499	0.1003	10.0333	0.0272	
7	1.2609	0.4174	0.1714	1.0916	2.2544	2121.5515	0.0987	9.8735	0.4174	
8	1.6992	0.5725	0.2244	0.7625	1.5668	1262.2314	0.1004	10.0404	0.5720	
9	-1.3916	-0.4640	0.2080	1.0739	2.2142	1951.2399	0.1001	10.0120	-0.4639	
10	1.0448	0.3469	0.1196	1.1030	2.2767	2120.4848	0.0993	9.9277	0.3469	
